# Enhanced Yield of Bioactivities from Onion (*Allium cepa* L.) Skin and Their Antioxidant and Anti-α-Amylase Activities

**DOI:** 10.3390/ijms21082909

**Published:** 2020-04-21

**Authors:** Mariana Gois Ruivo da Silva, Mihaela Skrt, Draženka Komes, Nataša Poklar Ulrih, Lea Pogačnik

**Affiliations:** 1 Department of Food Science and Technology, Biotechnical Faculty, University of Ljubljana, 1000 Ljubljana, Slovenia; marianagoisrs@gmail.com (M.G.R.d.S.); mihaela.skrt@bf.uni-lj.si (M.S.); natasa.poklar@bf.uni-lj.si (N.P.U.); 2 Department of Food Engineering, Faculty of Food Technology and Biotechnology, University of Zagreb, 10000 Zagreb, Croatia; dkomes@pbf.hr; 3 The Centre of Excellence for Integrated Approaches in Chemistry and Biology of Proteins, 1000 Ljubljana, Slovenia

**Keywords:** *Allium cepa* L., anti-α-amylase activity, antioxidant capacity, extraction optimization, quercetin

## Abstract

There is increasing concern for reduction of the ecological impacts of industrial waste caused by fruits and vegetables. To reduce costs of onion waste disposal while obtaining value-added products, onion skin can be used to extract quercetin, a natural flavonoid with antioxidant, anti-inflammatory and anti-cancer effects. The aim was to optimize quercetin extraction from brown onion (*Allium cepa* L.) skin through investigation of the effects of different parameters on quercetin yield. Operational parameters for conventional maceration extraction and for ultrasound-assisted extraction were compared: solvent type, mass-to-liquid ratio, extraction time and temperature. Antioxidant capacity was determined using DPPH· radical scavenging assays and quercetin yield using HPLC/DAD. Anti-α-amylase activity of onion skin extracts was investigated using α-amylase inhibition assays. Optimal extraction conditions of quercetin from onion skin were obtained with maceration extraction, 50% ethanol, 1:100 mass-to-liquid ratio, 25 °C, for 15 min. Under these conditions, the antioxidant capacity (expressed as quercetin equivalents) was 18.7 mg/g and the mass fraction of quercetin was 7.96 mg/g. The onion skin extracts showed a dose-dependent relationship between dry extract concentration and α-amylase inhibition, which confirms that this onion skin extract can be considered as an anti-diabetes agent.

## 1. Introduction

Brown onion, which is also known as yellow onion (*Allium cepa* L.), is a biennial herbaceous plant that originated from the territories of western and central Asia. In the European Union, 500,000 ton of onion waste is produced annually (comprising: stalk, skin, small and damaged onions), which represents an ecological problem [1]. However, onion skin can be used to extract its natural bioactive compounds, such as quercetin, a strong antioxidant of the flavonoids group [2]. Quercetin has beneficial effects on human health because of its antioxidant, anti-inflammatory, antimicrobial, antiviral, anti-allergic, cardioprotective, vasodilatory and anticancer activities [2,3,4]. It also stabilizes cell membranes, inhibits the aging process of the skin, cornea and myocardium, and has positive effects on the function of the cardiovascular system [5]. Quercetin is found in many medicinal plants and in fruit and vegetables [5], and it is known that the dry outer skin of brown onion is one of the richest sources of free quercetin [6]. Quercetin often occurs in nature not only in its free form, but also in the form of its glycosides, the most common of which is rutin [5].

Extraction procedures for quercetin and its glycosides from plant materials have been intensively developed and optimized in recent years [7]. The most common method of extraction in the literature is conventional maceration extraction (CME), as this does not require special equipment; however, it is time consuming and uses large solvent volumes [6,7,8,9]. The second most common method is ultrasound-assisted extraction (USAE), where the solid particles are vibrated under ultrasonic waves, to collapse the biologic membranes for the release of extractable compounds into the solvent. The solubility of quercetin in organic solvents has been shown to depend upon its amphipathic behavior [10]. It is therefore poorly soluble in water, and is instead soluble in ethanol [11] and methanol solutions, and in acetic acid and alkalis, among others [5]. An increased water fraction results in greater solubility of the more hydrophilic glucosides, whereas an increased ethanol fraction enhances the solubility of the more lipophilic aglycone. At the same time, some of the water of the aqueous fraction is necessary for effective swelling of the plant tissues during extraction, to increase the surface area for solid–solvent contact [12]. It is important to note that although ethanol is classified as a ‘generally recognized as safe’ solvent, its use in this application is restricted by the long extraction time and the strict legal statutes in many countries [6].

There have been several previous studies on the optimization of quercetin extraction from onion skin. Jin et al. (2011) [13] optimized various procedures, including CME, USAE and microwave-assisted extraction. The greatest quercetin yield for CME was obtained with 59.3% ethanol at 59.2 °C with 16.5 min of extraction. However, the most productive method was microwave-assisted extraction, in which the maximum extraction yield was 20.3% and 30.8% greater than those for USAE and CME, respectively. Jang et al. (2012) [12] investigated quercetin extraction using USAE and the optimal quercetin mass fraction was obtained with 59% ethanol (pH 2) with 1:60 mass-to-liquid ratio at 49 °C for 35 min. Savic-Gajic et al. (2018) [2] obtained optimal extraction conditions using 80% ethanol (pH 1.0) with a mass-to-liquid ratio of 1:64 for 47.3 min. Recently, Santiago et al. [14] performed the high-scale extraction of quercetin by incorporating a biorefinery approach, developing a full-scale plant for the valorization of onion solid waste into quercetin and fructooligosaccharides, under a circular economy perspective and, in parallel, evaluating the environmental profile of this alternative according to a life cycle assessment perspective. They concluded that the improvement alternatives should be studied (e.g., microwave and ultrasound-assisted extractions) to significantly reduce impacts on the environmental profile of this process.

Previous studies have related polyphenols, which include quercetin, to anti-α-amylase effects [15,16]. Diabetes mellitus type II is a chronic metabolic disorder caused by increased cell resistance to insulin. Benefits of pharmaceutical factors to treat this disease aggressively in its early stages were indicated, but such medications can have unwanted side effects. In this context, polyphenols (and thus quercetin) may be effective for the treatment of patients with diabetes mellitus type II due to their many hypoglycemic effects, including inhibition of α-glucosidase and α-amylase, which are key enzymes in the digestion of dietary carbohydrates into glucose. Through inhibition of these enzymes, polyphenols can delay carbohydrate digestion, which results in decreased glucose absorption, thereby reducing the postprandial plasma glucose rise [17,18].

Some of the previous studies [17,19,20,21,22], have also indicated a possible relationship between quercetin and anti-diabetes effects (either through α-amylase or α-glucosidase inhibition). Other studies showed possible anti-diabetes effects of onion by-products [23,24,25,26,27,28]. However, to the best of our knowledge, no studies of anti-α-amylase effects have been carried out with onion skin extracts in correlation with their quercetin content, optimized by a systematic study

The aims of this study were therefore: (i) to optimize the parameters of quercetin extraction from brown onion skin (*Allium cepa* L.) to thus maximize quercetin yield; and (ii) to determine the anti-diabetes properties of these extracts, based on the optimized procedure.

## 2. Results and Discussion

Four variables were studied here to optimize quercetin extraction through the primary and secondary extractions from onion skin: solvent, mass-to-liquid ratio, temperature and time. For each section below, the data from the 2,2-diphenyl-1-picrylhydrazyl radical (DPPH·) assays and the HPLC/ diode array detector (DAD) analyses are presented, with the data in all of Figures as means ±standard deviation. Table 1 gives a summary of the extraction conditions used for these optimizations.

### 2.1. Solvent Selection

The conditions for the selection of the optimal solvent for quercetin extraction from onion skin were as described in Section 3.3 and summarized in Table 1. The two extraction procedures of CME and USAE and the primary and secondary extractions were also compared here.

#### 2.1.1. Conventional Maceration Extraction

**Figure 1:**
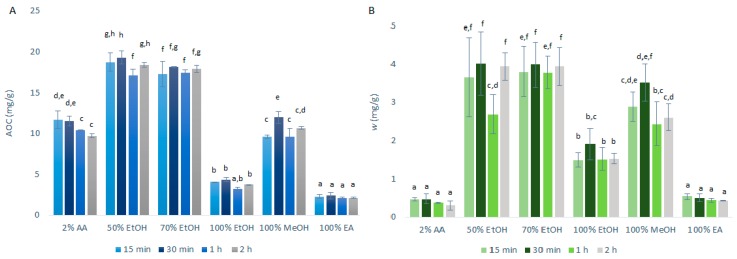
Quercetin equivalent antioxidant capacity (AOC) (**A**) and mass fraction (w) of quercetin (**B**) for the time courses of the primary extractions using conventional maceration with the different solvents at 1:10 mass-to-liquid ratio and 25 °C. Data are expressed as mg quercetin per g dry matter, as means ±standard deviation. Different letters within each histogram indicate significant differences (*p* < 0.05; ANOVA). AA, acetic acid; EtOH, ethanol; MeOH, methanol; EA, ethyl acetate.

The antioxidant capacity (AOC), expressed as quercetin equivalents was determined using DPPH· assays, along with the mass fraction of the quercetin obtained, as determined by HPLC/DAD. The data following the primary extraction with CME are shown in Figure 1. As can be seen from Figure 1A, the highest AOCs through the extraction time courses were seen for the 50% ethanol (17.1–19.3 mg/g) and 70% ethanol (17.3–18.1 mg/g) primary extracts. These were further decreased for 100% ethanol (3.2–4.4 mg/g) and were some 50% lower for 2% acetic acid (9.7–11.7 mg/g) and 100% methanol (9.6–12.0 mg/g). Particularly low AOCs were seen for 100% ethyl acetate (2.1–2.4 mg/g).

These data were largely expected as quercetin shows amphipathic behavior [10], as indicated in Section 1. This means that it will have a greater solubility in solvents that also display this behavior, such as ethanol/ water solutions. On the contrary, 2% aqueous acetic acid remains too polar as a solvent for quercetin and ethyl acetate too hydrophobic, which appears to justify the negative influence in the extraction yield of the polyphenols, including quercetin.

These data also show that the AOCs for most of the solvents selected are not strongly influenced by the duration of the extraction, although they were generally slightly improved from 15-min to 30-min. However, these differences did not reach statistical significance. Due to these data, the following USAE was performed with only 15-min and 30-min extractions.

As shown in Figure 1B, the data for the quercetin mass fraction in the dry matter follow a similar pattern to the AOC data. Greater primary extraction of quercetin was obtained for 50% ethanol (2.7–4.0 mg/g) and 70% ethanol (3.8–4.0 mg/g), whereas significantly less quercetin was obtained for 100% ethanol (1.5–1.9 mg/g), 100% ethyl acetate (0.43–0.53 mg/g) and 2% acetic acid (0.31–0.47 mg/g).

The correlation observed between the antioxidant capacity (as AOC; by DPPH· assays) and the amount of quercetin in the extracts (by HPLC/DAD) indicates that the DPPH· assay can be used as a screening test to evaluate and predict the extraction efficiency of quercetin. However, it can also be seen that this correlation between AOC and quercetin mass fraction was weaker for 2% acetic acid compared to the other solvents, which indicates that while the extraction with 2% acetic acid may be satisfactory for polar antioxidants, it is worse for the extraction of the less polar quercetin. This was as expected, as quercetin is poorly soluble in water [5], which is the main constituent of the 2% acetic acid solvent.

Following these primary extractions, the data for the AOCs (by DPPH· assays) and mass fractions of quercetin (by HPLC/DAD) were obtained after the secondary extractions with CME. Lower AOCs were determined in extracts obtained from the onion skin during the secondary extractions compared to the primary extractions, although the levels obtained were not negligible. As expected, the quercetin mass fractions obtained generally followed the same pattern as the AOCs.

The relative proportions of the AOCs and quercetin mass fractions determined for the primary and secondary extractions were also calculated. All of the secondary extractions still showed relatively high AOCs (secondary/ primary: 43–64%) and mass fractions of quercetin (secondary/ primary: 33–75%). This means that it was worthwhile not only to perform the primary extractions, but also to proceed with the secondary extractions, to extract more antioxidant activity and quercetin from the onion skin.

However, there are some notable differences among the solvents. For the quercetin mass fractions, the solvent with the highest remaining proportion of quercetin was 2% acetic acid (75% ± 12%), which was not the case for AOC (56% ± 10%). These data can be explained by the poor solubility of quercetin in water [5], which resulted in a high amounts of quercetin left after the primary extraction. Thus, the use of this two-stage extraction procedure to extract the most quercetin possible was more important for the 2% acetic acid extraction than for the 50% and 70% ethanol extractions.

#### 2.1.2. Ultrasonic Assisted Extraction

**Figure 2:**
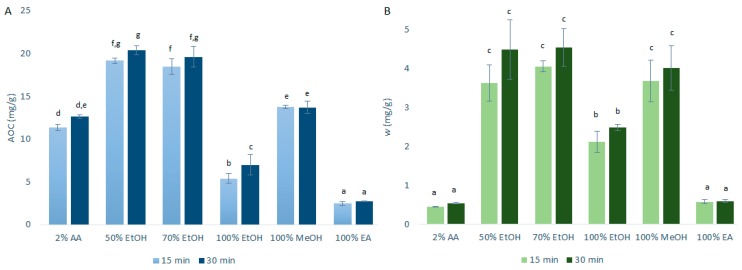
Quercetin equivalent antioxidant capacity (AOC) (**A**) and mass fraction (w) of quercetin (**B**) for the time courses of the primary extractions using ultrasound-assisted extraction with the different solvents at 1:10 mass-to-liquid ratio and 25 °C. Data are expressed as mg quercetin per g dry matter, as means ±standard deviation. Different letters within each histogram indicate significant differences (*p* < 0.05; ANOVA). AA, acetic acid; EtOH, ethanol; MeOH, methanol; EA, ethyl acetate.

The data for the AOCs (by DPPH· assays) and the quercetin mass fractions (by HPLC/DAD) obtained after the primary extraction with USAE are shown in Figure 2. As can be seen in Figure 2, the AOCs and the quercetin mass fractions for these primary onion skin extracts of 15 min and 30 min were a little higher with USAE compared to CME. These differences were more pronounced for the ethanol and methanol extractions. This was largely expected as USAE promotes greater agitation (cavitation) to generally improve the extraction efficiency compared to CME [8]. In addition, hence, as USAE generates an increased temperature that is hard to control, it is plausible that these small increases in the extraction yields were due to higher temperatures of the extractions rather than the use of USAE itself (although this hypothesis is denied by the data in the following sections). As these increased extraction yields with USAE were only a little higher than for CME, to reduce the costs associated with the USAE equipment and to avoid the potential lack of temperature control, it was decided to use CME for investigation of the further variables used here to optimize the quercetin extractions.

### 2.2. Mass-to-Liquid Ratio Selection

From the data in the Section 2.1., it was concluded that both 50% and 70% ethanol solutions represented the best extraction solvents. Considering the future applications of this extraction, it was considered that the lower the content of ethanol the better, to reduce costs and increase the process acceptability by reducing the health concerns regarding the final product. As indicated in Section 1, although ethanol is classified as a ‘Generally Recognized-as-Safe’ solvent, its use in this application is restricted by strict legal statutes in many countries [6]. Therefore, 50% ethanol was chosen as the optimal solvent for the further investigations.

**Figure 3:**
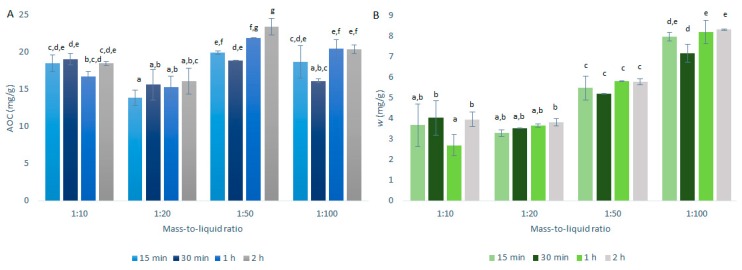
Quercetin equivalent antioxidant capacity (AOC) (**A**) and mass fraction (w) of quercetin (**B**) for the time courses of the primary extractions using conventional maceration with the different mass-to-liquid ratios and 50% ethanol at 25 °C. Data are expressed as mg quercetin per g dry matter, as means ±standard deviation. Different letters within each histogram indicate significant differences (*p* < 0.05; ANOVA).

The conditions for the selection of the optimal mass-to-liquid ratio for quercetin extraction from onion skin were as described in Section 3.3 and summarized in Table 1. The AOCs (by DPPH· assays) and the quercetin mass fractions (by HPLC/DAD) for the primary extractions using CME are shown in Figure 3.

As seen in Figure 3A, the best mass-to-liquid ratio for the AOCs was 1:50. This result appears reasonable, as the higher the volume of the solvent in relation to the mass of the solids, the greater the extraction of the antioxidants. However, there must be a balance, as lower amounts of solids will also mean lower total amounts of antioxidants present in the extractions.

**Figure 4:**
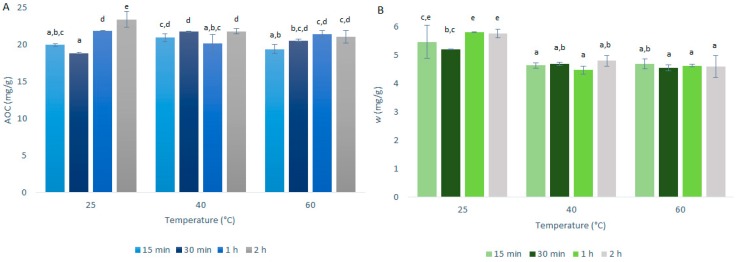
Quercetin equivalent antioxidant capacity (AOC) (**A**) and mass fraction (w) of quercetin (**B**) for the time courses of the primary extractions using conventional maceration with different temperatures and 50% ethanol and 1:50 mass-to-liquid ratio. Data are expressed in milligrams of quercetin per gram dry matter, as means ±standard deviation. Different letters within each histogram indicate significant differences (*p* < 0.05; ANOVA).

As seen in Figure 3B, the quercetin mass fraction distributions in these onion skin extracts relative to the mass-to-liquid ratios were different compared to the AOC distributions in Figure 3A. Essentially, the quercetin mass fractions increased with the increases in the solvent volume over the solids mass, thus resulting in the optimal extraction for the 1:100 mass-to-liquid ratio. These data suggest that quercetin requires a higher volume of solvent to solids mass to be efficiently extracted from onion skin, compared to the other antioxidants that showed higher levels (i.e., higher TAECs) with the 1:50 mass-to-liquid ratio (Figure 4A). During this study, the DPPH· assays were carried out before the HPLC analyses and were thus used as an indication of the HPLC results. Therefore, for the further investigation of the temperature selection, the 1:50 mass-to-liquid ratio was used as the optimal extraction ratio (i.e., for optimal extraction of all antioxidants by DPPH· assays).

### 2.3. Temperature Selection

The investigation of the optimal extraction temperature for quercetin extraction from onion skin was as described in Section 3.3 and summarized in Table 1. The AOCs (by DPPH· assays) and quercetin mass fractions (by HPLC/DAD) for the primary extractions using CME are shown in Figure 4.

As seen in Figure 4, the AOCs and quercetin mass fractions did not vary to any great extent with the primary extraction temperature; however, there were some differences between these two analyses. The AOCs across the three temperatures used were very similar, with the variations generally contained within the experimental standard deviation. In contrast, for the quercetin mass fractions, these were a little higher at 25 °C compared to the higher temperatures tested (40 °C, 60 °C). Indeed, it is well known that the solubility of quercetin in mixtures of ethanol increases smoothly with increasing temperature [13]. However, with the increase in temperature, the connections and structure of the quercetin may be destabilized, which would affect the quercetin mass fraction more specifically here, which resulted in its slight decrease with the higher temperatures.

These data confirm that the increase in the extraction yields for USAE compared to CME was indeed due to the method rather than an increase in temperature, as the increase in temperature from 25 to 40 °C did not result in increased extraction yield.

### 2.4. Optimized Quercetin Extraction

Across the parameters used as variables in this study, the variation in the solvent type had the highest impact, with larger significant differences in the yields of antioxidants and the quercetin mass fractions when compared to the remaining variables examined here. Therefore, the selection of solvent is the most important parameter to be defined when considering the extraction of quercetin from onion skin and this should be analyzed at the beginning of any new approach, as carried out for the present study. This is in agreement with the study of Jang et al. (2012) [12], who concluded that ethanol concentration and temperature are the most influential parameters compared to pH, mass-to-liquid ratio and extraction time.

In conclusion, focusing on the data for the quercetin mass fraction, the most efficient extraction was achieved with CME using 50% ethanol as the solvent at a 1:100 mass-to-liquid ratio. The optimal time was 15 min, as there were no great differences between the extractions of 15 min, 30 min, 1 h and 2 h; hence, the shortest time is the most economically feasible. The extraction temperature optimization showed that this was 25 °C, considering that the data for the 1:50 mass-to-liquid ratio were generally similar to those for the 1:100 mass-to-liquid ratio. Under these optimal extraction conditions, the AOC was 18.7 mg/g and the quercetin mass fraction was 7.96 mg/g.

The optimal extraction parameters obtained in this study are in agreement with those obtained by Jin et al. (2011) [13]. In their study, the highest quercetin yield for CME was obtained for 59.3% ethanol at 59.2 °C, with an extraction time of 16.5 min. These optimal parameters are similar to those obtained in the present study, except for the optimal temperatures. In the study by Jin et al. (2011) [13], the USAE data were also very similar to the CME data, which was also seen for the present study. Their quercetin yield for the conventional CME was 3.42 mg/g, which was less than that obtained in the present study.

The study by Jang et al. (2012) [12] investigated the extraction of quercetin with aqueous ethanol solutions from onion solid waste under sonication conditions (i.e., USAE). The quercetin mass fraction obtained in their study was 11.08 mg/g dry weight of onion solid waste, for their optimal conditions (59% ethanol, 49 °C), which represents a similar mass fraction to that obtained in the present study.

Another important parameter that has to be considered for optimal solvent choice is the quercetin retrieved in the dry extracts, as in large-scale applications of this process the extracts will be dried to remove the organic solvent. Therefore, the amounts of this bioactive compound in the extraction powder is of particular relevance here. As seen for the data given in Table 2, the mass of the dried extracts (6.8–30.3 mg) was considerably lower than the original mass of the dry matter used for the extraction (200 mg). This means that most of the solids in the original dry matter did not dissolve in any of the solvents but were discarded. The 50% ethanol extraction resulted in the most solids after drying, which corresponded to the optimal extraction determined by this study, in terms of solvent optimization.

Furthermore, comparisons of the quercetin mass fractions in the original dry matter (i.e., the lyophilized onion skin) and in the final dried extracts show that for all of these extraction solvents, these were considerably higher in the dry extracts. As such, this shows that these extraction procedures resulted in purer quercetin in the dry extracts compared to the original dry matter. The highest purification was for the 100% ethanol extraction (23-fold), while the 50% ethanol and 70% ethanol saw the quercetin purified by 6.6-fold and 9.1-fold, respectively. However, using 100% ethanol as extraction solvent is not environmentally friendly and there is a major restriction in many countries to use ethanol, which makes this approach impractical in a large scale. At the same time, the highest quercetin mass fraction in the dry extracts was determined for the 100% methanol extract (47.4 mg/g). However, considering the scalability of the process, not only is this parameter relevant, but also in particular the total mass of quercetin that can be retrieved from the dry extracts. The highest amounts of quercetin obtained from the extraction of 200 mg of lyophilized onion skin were seen for the 70% ethanol (0.344 mg), 50% ethanol (0.293 mg) and 100% methanol (0.260 mg) extractions.

As such—and considering the full data in the present study—the lower the content of ethanol in the extracts the better, to reduce extraction costs and to increase process acceptability by reducing health concerns regarding the final product. Therefore, the solvent of 50% ethanol can be considered as the optimal solvent for this quercetin extraction and its purity in the dry extract, further supporting the previous indication here for this optimal solvent.

Of note, the quercetin mass in the dried extract from the 50% ethanol extraction has further relevance below for the comparison of the anti-α-amylase data obtained for this extract, in terms of the quercetin that it contained.

### 2.5. Anti-α-Amylase Activity

**Figure 5:**
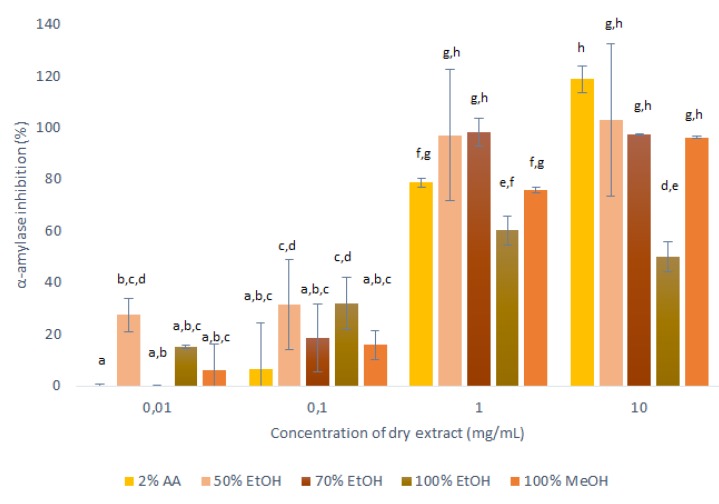
Anti-α-amylase activities of the dry matter from the primary extractions using conventional maceration with the different solvents (1:10 mass-to-liquid ratio; 25 °C; 15 min), in terms of inhibition of α-amylase activity. Data are means ±standard deviation, and the different letters indicate significant differences (*p* < 0.05; ANOVA). AA, acetic acid; EtOH, ethanol; MeOH, methanol.

As described in Section 3.6, to determine the potential anti-α-amylase activities of the dried extracts, these were then resuspended at a range of concentrations for testing in α-amylase assays, as shown in Figure 5.

These data for the inhibition of α-amylase showed dose-dependent relationships for all of these extracts: the higher the concentration of the applied extract, the higher the inhibition of α-amylase. However, considerable differences across these extracts were seen for the different solvents. The extracts obtained with 50% ethanol, 100% ethanol and 100% methanol decreased the α-amylase activity across all of the concentrations tested, while the 2% acetic acid and 70% ethanol extracts showed no anti-α-amylase effects at the lowest concentrations tested (0.01 mg/mL).

The extracts obtained with 50% ethanol and 70% ethanol appeared to completely inhibit α-amylase already at 1 mg/mL, and for the 100% ethanol extract the inhibition did not increase for the highest tested concentration (10 mg/mL), which indicated that its maximum inhibition was reached already at 1 mg/mL (60%). However, the trend of increasing inhibition with higher concentration was observed for 2% acetic acid extracts during all tested concentrations of dry extract. Therefore, at the highest concentration of these extracts (10 mg/mL), the α-amylase activity was completely inhibited by all of the solvents except for 100% ethanol, which appeared to be the worst extraction solvent for substances with anti-α-amylase activity.

It can thus be concluded that in general all onion skin extracts are promising inhibitors of α-amylase, and can, therefore, be considered as anti-diabetes agents. The optimal solvent regarding α-amylase inhibition was 50% ethanol here. This is in agreement with the AOC data as for the mass-to-liquid ratio of 1:10 used in these assays, 50% ethanol was considered the optimal solvent (with the highest AOC). This high value of antioxidants can be assumed to be due to the high polyphenol contents of the extract, which can interact with the α-amylase to result in the highest inhibition.

The conclusion that onion skin extracts can be considered as anti-diabetes agents is here partly based in previous studies that have shown anti-α-amylase activities for polyphenols [17,19]. However, the data in the present study demonstrate that these onion skin extracts that contain quercetin have a direct impact on the α-amylase activity and pointing out the fact that other substances, present in these extracts may have important impact on the overall anti-α-amylase activity, which was not shown by other studies. These data reinforce the possibility of using onion skin extracts in the human diet and as a food supplement, as an alternative in the management of diabetes.

### 2.6. Impact of the Quercetin in Onion Skin Extracts

The goal was then to determine the actual impact that the quercetin in these extracts has compared to other compounds they may contain, in terms of the anti-α-amylase activity. For this, the α-amylase assay was also carried out with pure quercetin, following the anti-α-amylase activity procedure as described in Section 3.6. The concentrated extracts for the sample analysis had a dry extract concentration of 10 mg/mL. The concentration of the stock solution of the quercetin standard that mimicked the quercetin concentration in this extract was calculated according to Equation (1), using the above-determined quercetin concentration in the dried extract of 24.3 mg/g for the selected solvent of 50% ethanol.

(1) $$0.0243\;\left(\frac{\mathrm{mg}\;\mathrm{quercetin}}{\mathrm{mg}\;\mathrm{dry}\;\mathrm{extract}}\right)\times 10\;\left(\frac{\mathrm{mg}\;\mathrm{dry}\;\mathrm{extract}}{\mathrm{mL}\;\mathrm{solvent}}\right)=0.243\left(\frac{\mathrm{mg}\;\mathrm{quercetin}}{\mathrm{mL}\;\mathrm{solvent}}\right)$$

**Figure 6:**
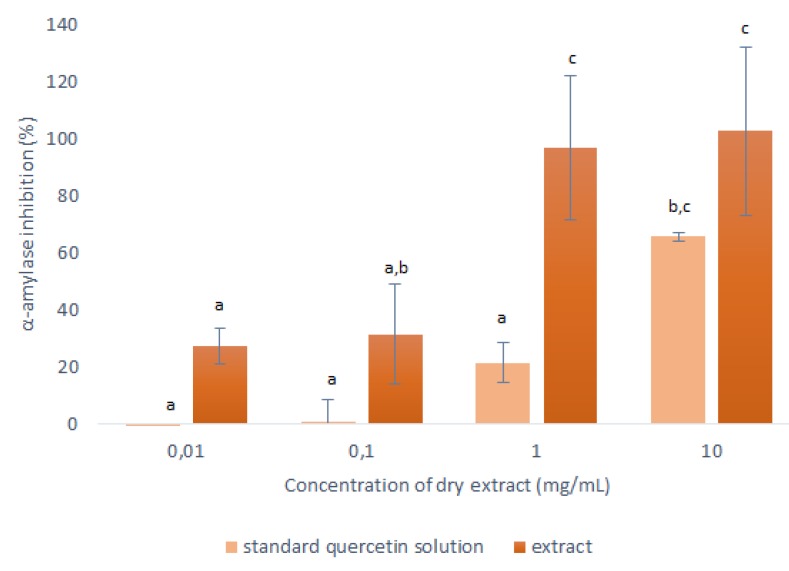
Anti-α-amylase activities of the dry matter from the concentrated 50% ethanol extracts (1:10 mass-to-liquid ratio, 25 °C; 15 min) and the corresponding stock solution of the quercetin standard, in terms of inhibition of α-amylase activity. Data are means ±standard deviation and different letters indicate significant differences (*p* < 0.05; ANOVA).

Therefore, the concentration of the stock solution of the quercetin standard was 0.243 mg/mL, which corresponded to the concentration of quercetin in the concentrated 50% ethanol extract at 10-mg/mL dry extract. This stock solution of the quercetin standard was diluted 10-fold, 100-fold and 1000-fold, to mimic the concentrated extracts with dry extract concentrations of 1 mg/mL, 0.1 mg/mL and 0.01 mg/mL, respectively. The data for their inhibition of α-amylase are shown in Figure 6.

As seen in Figure 6, the α-amylase inhibition was consistently lower for the quercetin standard compared to the corresponding concentrated onion skin extract. This was as expected, as it shows that the extracts contained other substances that can also interact with the α-amylase.

The most relevant data here were obtained at 1 mg/mL, where the responses of the pure quercetin and the onion skin extract were significantly different. Here, 0.0243 mg/mL of the quercetin standard showed 22% inhibition of the α-amylase activity; therefore, in the equivalent onion skin extract (containing 0.0243-mg/mL quercetin), 78% of its inhibition can be attributed to other substances in the extract. These data indicate that the use of this onion skin extract that contains a complete range of polyphenols should be more beneficial in terms of anti-α-amylase activity than using pure quercetin. Further studies focused on identification of other substances in onion skin extracts with antioxidant and anti-α-amylase activity have to be performed in order to clarify the total effect of these extracts.

## 3. Materials and Methods

### 3.1. Chemicals

DPPH·, Formic acid (>98%) and quercetin (>95%) were from Sigma Aldrich (Darmstadt, Germany). Ethyl acetate (99.5%), acetonitrile (>99.9%) and ethanol (96%) were from Honeywell (Riedel-de Haen, Germany). Absolute ethanol and absolute methanol were from Emsure, Merck (Darmstadt, Germany). Sodium hydroxide, sodium potassium tartrate tetrahydrate and maltose were from Kemika (Zagreb, Croatia). Dinitrosalicylic acid (DNSA) and soluble starch were from Merck (Darmstadt, Germany) and α-amylase from hog pancreas (43.6 U/mg) was from Fluka (Buchs, Switzerland).

### 3.2. Plant Materials

The skin from brown onions (*Allium cepa* L.) was collected in February 2019 from Celje, Slovenia. The skin was weighed (Exacta 2200 EB; Tehtnica, Slovenia) in beakers and frozen with liquid nitrogen before freeze-drying in a lyophiliser (Alpha 1–2 LD Plus; Christ, Germany) at −50 °C and 0.12 mbar, for 3 days. The beakers were then weighed again to obtain the mean proportion of evaporated water. The dried samples were powdered using an analytic mill (A11 Basic; IKA, Germany) and stored in the freezer (−20 °C) until use. All of the data are expressed per g freeze-dried onion skin obtained by this procedure (defined as /g dry matter).

### 3.3. Extraction Procedures

The initial aim here was to determine the optimal solvent for the extraction of quercetin from onion skin. For this, a mass-to-liquid ratio of 1:10 was obtained by adding 1 mL of the following solvents to 100 mg of the powdered samples (see Section 3.2.) in test tubes: 2% acetic acid, 50% ethanol, 70% ethanol, 100% ethanol, 100% methanol and 100% ethyl acetate. The samples underwent the primary extraction by CME by agitation in a thermo-shaker (TS1; Biometra, Germany) at 25 °C for 15 min, 30 min, 1 h and 2 h. They also underwent the primary extraction by USAE in an ultrasonic bath (Ultrasonic Cleaner 100 W; Shesto, UK) at the highest setting at 25 °C for 15 min and 30 min. It was not possible to closely control the temperature in the ultrasonic bath, and therefore there was an estimated increase of 5 °C to 10 °C from the stated temperature. The samples were centrifuged (Centric 200; Tehtnica, Slovenia) at a relative centrifugal force of 25,230× *g* for 5 min, and the solvent was removed with a pipette and stored in a freezer (−20 °C) until use.

Secondary extractions were also performed, by addition of 1 mL of the relevant solvent to each sample in the test tubes that contained the remaining solid material after the primary extraction. This process was repeated as described above, with the primary and secondary extractions maintained separate.

The next aim was to select the optimal mass-to-liquid ratio. For this, three additional mass-to-liquid ratios were tested as well, as 1:20, 1:50 and 1:100. For each mass-to-liquid ratio, 1 mL 50% ethanol solution was added to 50 mg, 20 mg and 10 mg dry matter, respectively, in the test tubes, as above. CME and USAE were carried out for these samples at 25 °C for 15 min, 30 min, 1 h and 2 h, only for the primary extractions.

The final aim was to select the optimal extraction temperature. For this, only CME was performed with two additional temperatures tested as well, as 40 °C and 60 °C, for 15 min, 30 min, 1 h and 2 h, with only the primary extractions with 50% ethanol and 1:50 mass-to-liquid ratio used.

Each optimization series was carried out as two independent repetitions, to access the consistency of the data.

For the anti-α-amylase activity assays (see Section 3.6), the dry extracts were also prepared using the samples from the solvent optimization with the mass-to-liquid ratio of 1:10 at 25 °C, and with 15 min extractions. All of these solvent samples were dried in a vacuum concentrator centrifuge (Univapo 100 H; Uniequip, Germany) for 3 h to 6 h, until completely dried.

### 3.4. Determination of Antioxidant Capacity

The AOCs were determined using the free radical scavenging DPPH· assay, which is based on determination of the capacity of a sample to neutralize the stable free radical DPPH· [29].

A calibration curve with 2% acetic acid was obtained in duplicate, using sample solutions of different quercetin concentrations prepared with 5 µL to 50 µL of 1.10-mM quercetin, supplemented with 2% acetic acid to 50 µL, with the control sample of 50 µL 2% acetic acid. The samples were treated with 1 mL 0.11-mM DPPH·, mixed and incubated at room temperature in the dark for 1 h. The absorbances were then measured at 517 nm using a UV-VIS spectrophotometer (89090A; Agilent, Santa Clara, CA, USA), with 2% acetic acid as the blank.

An additional calibration curve for the extracts with ethyl acetate was prepared, due to the different responses of this solvent. This used 20 µL ethyl acetate and 5 µL to 30 µL 1.10-mM quercetin and was supplemented with 2% acetic acid to 30 µL, with the control sample containing 20 µL ethyl acetate and 30 µL 2% acetic acid. The remaining procedure was conducted as described above.

The extracts were diluted accordingly for each assay, and 50 µL of diluted extracts was mixed with 1 mL 0.11-mM DPPH·. The samples were analyzed as two independent repetitions and treated as described above for the calibration curve procedures. The data are expressed as mg of quercetin equivalent per g dry matter (AOC).

### 3.5. Quantification of Quercetin

Quantification of quercetin was achieved using a HPLC/DAD system (1260 Infinity; Agilent, USA) which included a binary pump (G1312B; Agilent), a vacuum degasser (G1322A; Agilent), a thermostated autosampler (G1367E, HiPALS; Agilent), a thermostat for the column (G1316A, TCC; Agilent) and a DAD (G4212B; Agilent). For the analysis, a C18 column was used (Zorbax Eclipse Plus; Agilent; inner diameter, 4.6 mm; length, 150 mm; particle size, 3.5 µm), which was connected to a precolumn (Zorbax Eclipse XBD-C18; Agilent; inner diameter, 4.6 mm; length, 12 mm; particle size, 5 µm) [30]. The autosampler temperature was set to 10 °C, the column temperature was set to 35 °C, the injection sample volume was 20 µL, and the flow rate was set to 0.8 mL/min.

The solvent system was composed of 0.1% formic acid in water (A) and acetonitrile with 0.1% formic acid (B), with the following gradient elution: equilibrated at 25% B; 0 → 10 min, 25% → 30% B; 10 → 20 min, 30% → 65% B; 20 → 21 min, 65% → 100% B; 21 → 22 min, 100% B; 22 → 23 min, 100% → 25% B; 23 → 27 min, 25% B (re-equilibration). Data were acquired using the HPLC 2D Chemstation software, revision B.04.03 (Agilent).

A mobile phase solution was prepared with 150 mL solution A plus 50 mL solution B (i.e., 25% B), to dilute the samples for the analysis. A stock solution of 0.9-mg/mL quercetin was obtained from 45.09 mg quercetin dissolved in 50 mL 100% ethanol. The calibration curve was constructed using 15 solutions with 0.15 µg/mL to 180 µg/mL quercetin, using different volumes of the 0.9-mg/mL quercetin stock solution supplemented with mobile phase solution to 1 mL. The samples were analyzed by HPLC/DAD and the chromatograms of the samples were recorded from 254 nm to 400 nm, with quercetin determined at 370 nm.

Dilutions of onion skin extracts of 1:4 to 1:40 were prepared using the mobile phase solution to obtain signal responses within the calibration curve. The samples were then centrifuged (25,230× *g* for 5 min) to remove non-soluble particles before the analysis. The data of the mass fractions of quercetin (*w*) are expressed as mg quercetin per g dry matter.

### 3.6. Determination of Anti-α-Amylase Activity

The anti-α-amylase activity was assessed using *α*-amylase inhibition assays, which measure the concentrations of reducing sugars spectrophotometrically at 540 nm using DNSA as the color reagent [31]. α-Amylase catalyzes the hydrolysis of α-1,4-glycosidic linkages of starch components to produce oligosaccharides, such as maltose [32].

The calibration curve was prepared from 1.8-mg/mL maltose stock solution, using 10 dilutions from 0.25- to 2.5-µmol/mL maltose. Four hundred microliters of each dilution was pipetted into each test tube, together with a control test tube with 400 µL distilled water. The test tubes were prepared in duplicate, and after a pre-incubation at 25 °C in a dry bath heating system (Star Lab; Taiwan), 200 µL DNSA color reagent was added to each test tube. These test tubes were then incubated in a boiling water bath for 15 min, and then cooled in ice. The reaction mixture was then diluted by adding 1.8 mL distilled water, following which the absorbance was measured at 540 nm.

The extracts analyzed in this assay (i.e., samples from solvent optimization with 1:10 mass-to-liquid ratio and 15 min extraction) were dried following the procedure described in Section 3.3 and resuspended in the respective solvents to obtain the same final concentration, of 10 mg dry extract per mL solvent. This was repeated for the samples of the two repetitions and the extracts were mixed.

Four concentrations of dry extract were prepared for each sample (0.01, 0.1, 1.0, 10 mg/mL). The three lower concentrations were prepared by serially diluting the 10-mg/mL dry extract in 20-mM sodium phosphate buffer, pH 6.9, 6-mM sodium chloride (SPB). The test tubes were prepared in duplicates with 100 µL of each concentration and 100 µL 0.1-mg/mL α-amylase. Each test tube had a corresponding control (without enzyme) with 100 µL of each sample and 100 µL SPB. Another control that corresponded to 100% α-amylase activity was also prepared in duplicate by adding 100 µL SPB and 100 µL 0.1-mg/mL α-amylase. The blank for this control was 200 µL SPB.

All of the test tubes and the substrate of 1% starch were equilibrated for 15 min at 25 °C. Then, 200 µL 1% starch was added to each test tube at 5 s intervals. The mixtures were incubated for exactly 3 min and then 200 µL DNSA color reagent was added, also at 5 s intervals, to stop the reaction. The procedure was continued as described for the calibration curve. The concentration of maltose in each tube was obtained and the inhibition of α-amylase activity was obtained as percentages according to Equation (2).

(2) $$\%\;\mathrm{inhibition}=\frac{c_{100\%\;\mathrm{activity}}-c_{\mathrm{sample}}}{c_{100\%\;\mathrm{activity}}}$$

where *c_100%_*_activity_ is the concentration of maltose in the test tube for 100% activity of *α*-amylase and *c*_sample_ is the concentration of maltose in the samples.

### 3.7. Statistical Analysis

The data are presented as means ±standard deviation across the two replicates. Statistical analysis was carried out using one-way analysis of variance (ANOVA), followed by Duncan’s post hoc tests, to define any significant differences between group means, using IBM SPSS Statistics for Windows (version 21, Armonk, NY, USA). Differences were considered significant at the *p* < 0.05 level.

## 4. Conclusions

Onions are the second most important horticultural crop worldwide, after tomatoes, with current annual production almost 100 million tons. Over the past 10 years, onion production has increased by almost 30% [33], leading to the increased amounts of waste, particularly onion skin that is presenting an environmental problem due to its characteristic aroma, which makes it unusable in high concentrations for fodder.

The optimal extraction conditions for quercetin from onion skin according to the quercetin mass fraction determined by this study, were obtained using CME with 50% ethanol as solvent, 1:100 mass-to-liquid ratio, 15 min extraction time and 25 °C extraction temperature. Under these conditions, the antioxidant capacity expressed as AOC was 18.7 mg/g; the quercetin mass fraction was 7.96 mg/g. This quercetin mass fraction is in agreement with some previous studies in this field [12,13]. It was also shown that all of the extraction procedures resulted in purer quercetin compared to the initial dry matter (i.e., lyophilised onion skin).

The optimal solvent results are in agreement with previous studies as it is generally accepted that aqueous-based ethanol solutions are the optimal solvents. It is important to note that both 50% and 70% ethanol were deemed to be optimal solvents; however, considering the future applications of this extraction, to reduce possible costs and to increase the health acceptability, the solvent of 50% ethanol was chosen as optimal. However, this evaluation remains to be carried out for each process while considering the actual economic and regulatory parameters.

For the comparisons of the primary and secondary extractions, it appears worthwhile to not only perform the primary extraction, but to also proceed with a secondary extraction to extract more antioxidants and quercetin from the onion skin. The comparisons of CME and USAE showed that both the AOCs and the quercetin mass fractions in these onion skin extracts were a little higher using USAE rather than CME, but considering the small differences here and the higher costs associated with USAE, CME is the better choice.

For the anti-α-amylase activities, all of the extracts analyzed showed dose-dependent relationships between dry extract concentration and α-amylase inhibition, which confirms the hypothesis that onion skin extracts containing quercetin can be used as anti-α-amylase agents. In addition, these data on the impact of quercetin showed that these onion skin extracts have improved anti-α-amylase potential than the corresponding quantities of pure quercetin. This result provides a further contribution to previous studies that have demonstrated other effects of quercetin in diabetes and confirms the potential of these onion skin extracts as an alternative therapy against diabetes. However, animal studies on their in vivo effects on α-amylase and their safety and potential dosing in humans are still required. Further studies on quercetin recovery and purification from ethanol extracts of onion skin also need to be carried out as well as these required in vivo studies, to determine the quercetin bioavailability and dosing with such extracts and also to investigate the impact of excess quercetin on the human body, before such extracts can be applied in the food supplement and food industry markets.

Finally, we believe that extraction of bioactivities with this optimized procedure, suggested by this study could not only reduce the amount of unwanted onion byproducts, but also enable production of a high value product, namely food supplement or nutraceutical, that could in the future be used both as a source of antioxidants as well as possible anti-diabetes agent. Consequently, the production costs due to waste disposal of onion production would be substantially reduced. However, during the production of extracts, the costs of extraction procedure (e.g., solvents, energy) has to be considered as well. These can very much be reduced by our proposed optimized extraction procedure, performed at low temperature (25 °C), short time (15 min) and low amount of organic solvent (50% ethanol).

## Figures and Tables

**Table 1:** Optimization of the conditions used for the primary and secondary extractions. See Section 3.3. for further details.

Optimization	Solvent	Mass-to-Liquid Ratio	Temperature (°C)	Time	
				CME	USAE
Solvent	2% acetic acid	1:10; fixed	25; fixed	15, 30 min; 1, 2 h,	15, 30 min
	50% ethanol				
	70% ethanol				
	100% ethanol				
	100% methanol				
	100% ethyl acetate				
Mass-to-liquid ratio ^a^	50% ethanol; fixed	1:10 ^b^	25; fixed	15, 30 min; 1, 2 h	15, 30 min; 1, 2 h
		1:20			
		1:50			
		1:100			
Temperature ^a^	50% ethanol; fixed	1:50; fixed	25 ^c^	15, 30 min; 1, 2 h	—
			40		
			60		

^a^, primary extractions only; ^b^, using the data from solvent optimization of 50% ethanol; ^c^, using the data from mass-to-liquid ratio optimization of 1:50; CME, conventional maceration extraction; USAE, ultrasound-assisted extraction.

**Table 2:** Quercetin recoveries obtained from 200 mg of lyophilized onion skin (original dry matter) according to the solvent optimization conditions (1:10 mass-to-liquid ratio; 25 °C; 15 min).

Extraction Solvent	Total Dry Extract (mg)	Quercetin		
		Mass Fraction		Final Mass (mg)
		Dry Matter (mg/g)	Dry Extract (mg/g)	
2% acetic acid	22.5	0.47 ± 0.05	4.2 ± 0.4	0.019 ± 0.002
50% ethanol	30.3	3.7 ± 1.0	24.3 ± 6.8	0.293 ± 0.082
70% ethanol	22.0	3.8 ± 0.7	34.8 ± 6.0	0.343 ± 0.060
100% ethanol	6.82	1.5 ± 0.1	35.9 ± 3.1	0.122 ± 0.011
100% methanol	12.3	2.9 ± 0.4	47.4 ± 6.3	0.260 ± 0.035

Data are means ± standard deviation.
